# Autophagy and its therapeutic potential in diabetic nephropathy

**DOI:** 10.3389/fendo.2023.1139444

**Published:** 2023-03-20

**Authors:** Yu-Peng Han, Li-Juan Liu, Jia-Lin Yan, Meng-Yuan Chen, Xiang-Fei Meng, Xin-Ru Zhou, Ling-Bo Qian

**Affiliations:** School of Basic Medical Sciences & Forensic Medicine, Hangzhou Medical College, Hangzhou, China

**Keywords:** diabetic nephropathy, autophagy, nutrient-sensing pathway, cellular stress, renal cell

## Abstract

Diabetic nephropathy (DN), the leading cause of end-stage renal disease, is the most significant microvascular complication of diabetes and poses a severe public health concern due to a lack of effective clinical treatments. Autophagy is a lysosomal process that degrades damaged proteins and organelles to preserve cellular homeostasis. Emerging studies have shown that disorder in autophagy results in the accumulation of damaged proteins and organelles in diabetic renal cells and promotes the development of DN. Autophagy is regulated by nutrient-sensing pathways including AMPK, mTOR, and Sirt1, and several intracellular stress signaling pathways such as oxidative stress and endoplasmic reticulum stress. An abnormal nutritional status and excess cellular stresses caused by diabetes-related metabolic disorders disturb the autophagic flux, leading to cellular dysfunction and DN. Here, we summarized the role of autophagy in DN focusing on signaling pathways to modulate autophagy and therapeutic interferences of autophagy in DN.

## 1 Introduction

Diabetic nephropathy (DN), a major cause contributing to end-stage renal disease (ESRD), is one of the microvascular complications of diabetes and is commonly rendered by persistent hyperglycemia and the subsequent chronic inflammatory response (1, 2). Almost 35%-40% of diabetic patients finally lead to DN (3), which poses a huge number of diabetic death and a serious threat to the quality of life in diabetes (4). International Diabetes Federation (IDF) Diabetes Atlas (the 10^th^ edition) showed that the number of adult diabetes worldwide will increase from 537 million in 2021 to 643 million by 2030 and over 6.7 million diabetes aged 20-79 years died from diabetes-related diseases in 2021 (http://diabetesatlas.org/atlas/tenth-edition/). Long-term diabetes can damage many organs to cause disabling and life-threatening complications including cardiovascular diseases, neuropathy, and nephropathy. DN, with clinical manifestations including progressive proteinuria as well as decreased glomerular filtration rate (3), and pathological features such as glomerular hypertrophy, glomerular basement membrane (GBM) thickening, mesangial proliferation, and podocyte loss (5), is one of the early complications in diabetes. Though keeping blood pressure, blood glucose, and the renin-angiotensin system (RAS) under control is a primary therapy to relieve proteinuria in diabetes, treatment-resistant proteinuria and ESRD have not been fully avoided (6). Exploring the underlying mechanism of DN and finding novel targets to effectively prevent DN have become urgent for improving the quality of life in diabetes.

The pathogenesis of DN is multifactorial (4), including oxidative stress, inflammatory cascade reaction, and other disorders of metabolic pathways under persistent hyperglycemia (7). Growing evidence reveals that along with diabetes, the accumulation of damaged organelles and proteins owing to impaired autophagy has been reported to disrupt cellular homeostasis and result in the development of DN (3, 7–10). Autophagy normally is activated to degrade impaired organelles or misfolded proteins as a recycling response to nutrition deprivation or starvation (10). The metabolic disorder manifested as persistent high blood glucose and lipids causes a state of overnutrition and suppresses autophagy in diabetic renal cells (11–13), while promoting autophagy lessens renal injury in diabetes (14, 15). All these clues suggest that activating autophagy may be a novel therapeutic target to prevent DN and shed light on treating DN based on the balance of autophagy.

Although the relationship between autophagy and DN has not been fully clarified, numerous studies have confirmed that the development of DN is linked to autophagy. Detailed exploration of autophagy in the pathogenesis of DN can provide new ideas for preventing DN. Thus, this review aims to understand the cellular and molecular bases of autophagy, the role of autophagy in the development of DN, and therapeutic strategies targeting autophagy for the prevention of DN by summarizing current evidence.

## 2 Profile of autophagy in DN

Autophagy is a highly conserved cellular mechanism by which cytoplasmic constituents including proteins and organelles are transported to lysosomes for degradation and preserving cellular homeostasis (9, 16). Basal cellular autophagy is necessary for keeping physiological functions, whereas autophagy in response to stress serves as an adaptive reaction to ensure cell survival (16). Autophagy is a multistep process that involves the formation of isolation membrane, extension, formation of autophagosome, and final fusion with lysosomes to degrade phagocytic materials and is regulated by multiple protein kinase complexes and autophagy-related proteins, such as autophagy-related gene 5 (Atg5), Atg7, Atg12 and so on (8, 17). Among them, activation of the unc-51-like kinase 1 (ULK1) complex is responsible for the initiation of autophagy (3, 10). The class III phosphatidylinositol 3-kinase (PI3K) complex generates phosphatidylinositol 3-phosphate at the neogenetic autophagosomal membrane to facilitate phagophore nucleation (18). Two ubiquitin-like coupling systems, Atg5-Atg12-Atg16L and Atg8/microtubule-associated protein 1A/1B-light chain 3 (LC3) are involved in autophagosome extension and autolysosome formation (19). Atg4 cleaves LC3 to form cytosolic LC3I, which is then ubiquitinated by Atg7 and Atg3 and binds to phosphatidyl ethanolamine to form autophagosome membrane-bound LC3II (17). Thus, LC3II is evidenced as a marker for autophagosome formation in cells. This conjugated response of LC3II is positively regulated by Atg5-Atg12-Atg16L. Sequestosome 1, known as p62, interacts with LC3II to confine autophagosomes and is repeatedly digested by the autophagy-lysosome system. Significantly, malfunctional autophagy during diabetes causes intracellular accumulation of p62 leading to further inhibition of autophagic flux, thus forming a vicious cycle to promote diabetic complications including diabetic cardiomyopathy, diabetic peripheral neuropathy and DN (20–22).

Autophagy can be triggered by various intracellular stresses, such as reactive oxygen species (ROS), endoplasmic reticulum (ER) stress, and hypoxia (23–25), all of which are involved in the development of DN. Increasing evidence indicates that the abnormal alteration of autophagy appears to be directly linked to the emergence of DN (26, 27). Autophagy is closely associated with nutrient-sensing signal pathways and stress metabolism and is essential to maintain homeostasis in the kidney (3). Although the mechanism of autophagy in DN remains to be elucidated, it has been known that the impaired autophagy is evidenced by the increased collection of p62 and the decreased expression of autophagy-related proteins in diabetic kidney tissues and cells (28–30). The shortage of autophagy results in the accumulation of misfolded or aging proteins and dysfunctional organelles to deteriorate kidney disease in diabetes (19). Activation of autophagy alleviates kidney lesions in diabetes (31, 32) while inhibition of autophagy worsens these diabetic injuries (33, 34), indicating that autophagy might be a promising therapeutic target for DN.

## 3 Autophagy in renal cells during diabetes

**Figure 1:**
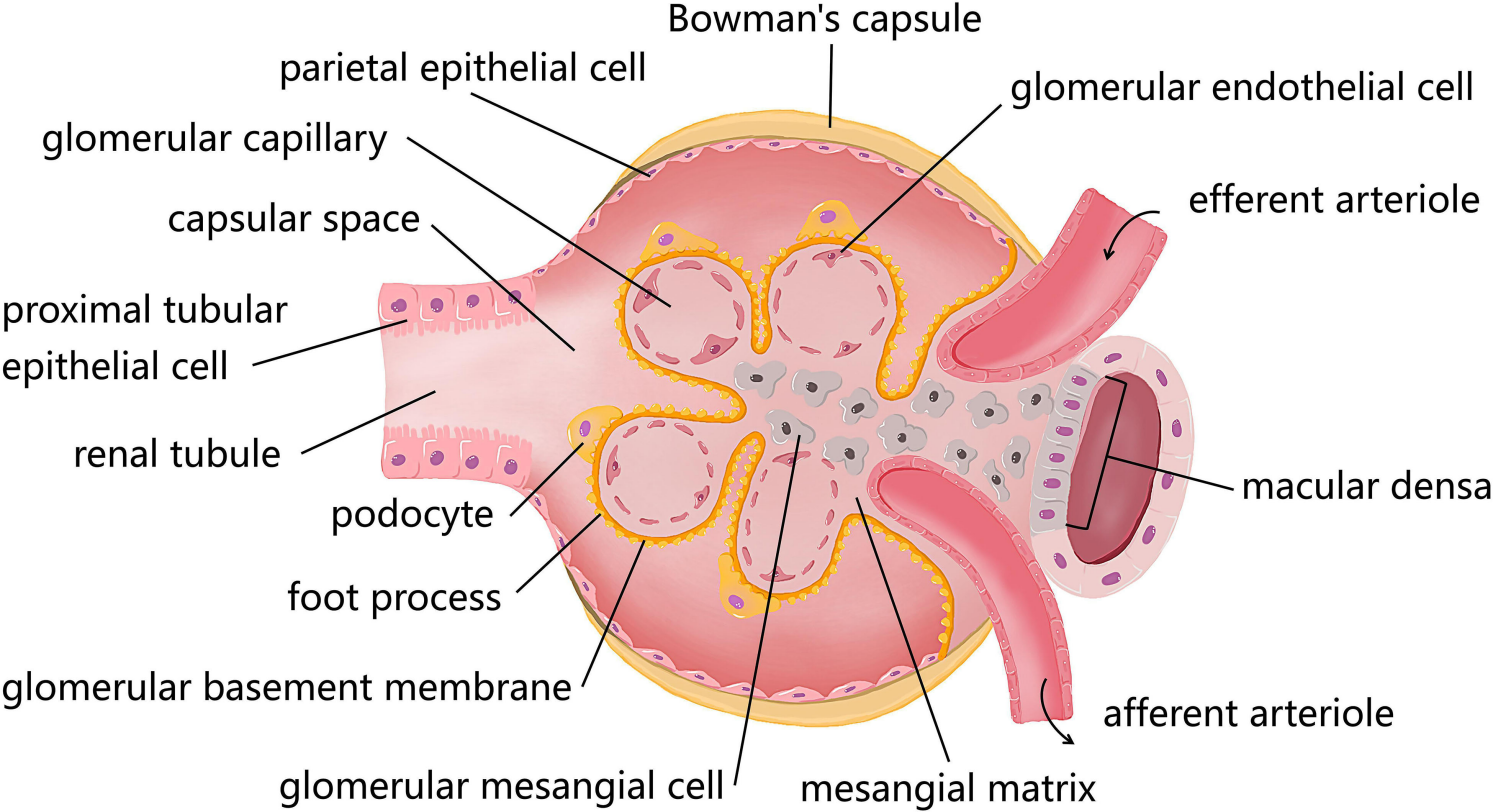
The diagram of resident cells in the glomerulus and proximal tubule. There are four kinds of major resident cells in the glomerulus, glomerular endothelial cells (GEnCs), podocytes, glomerular mesangial cells (GMCs), and parietal epithelial cells. Tubular epithelial cells form the extension of Bowman’s capsule, that is, the renal tubule. Podocytes with their interdigitating foot processes are arranged on the lateral side of the glomerular basement membrane (GBM). GMCs located between glomerular capillary loops, adjacent to endothelial cells or basement membranes are irregularly shaped. GEnCs are flat cells attached to the GBM. GEnCs and podocytes form the glomerular filtration barrier.

Though different types of renal cells are all damaged by the dysfunctional autophagy in the progression of DN, as shown in **Figure 1**, these four resident renal cells including podocytes, renal tubular epithelial cells (RTECs), glomerular mesangial cells (GMCs), and glomerular endothelial cells (GEnCs) may be particularly vulnerable to attack from the disorder of autophagy and contribute to DN. Thus, we summarized recent findings of renal cells in diabetic environments to better understand autophagy in DN (**Table 1**).

**Table 1:** Autophagy in four types of renal cells during diabetes.

Cell types	Major findings
Podocytes	• Silence of miR-150-5p attenuates DN by targeting Sirt1/p53/AMPK-dependent autophagy (12) and suppression of miR-383-5p alleviates high glucose-induced apoptosis *via* the activation of autophagy (35), while miR-25-3p attenuates high glucose-induced injury through suppressing dual specificity protein phosphatase 1 and subsequently activating autophagy in podocytes (36). • Promotion of autophagy by inhibiting Akt/mTOR pathway protects the DN serum-treated or high glucose-treated podocytes against apoptosis (34, 37, 38). • Activation of epidermal growth factor receptor in podocytes contributes to progression of DN partly caused by up-regulating rubicon and inhibiting the subsequent autophagy (39). • Regulating Bcl-2-mediated crosstalk between autophagy and apoptosis attenuates podocytes injury in diabetes (40). • Activation of AMPK and Sirt1-mediated autophagy ameliorates lipid accumulation, oxidative stress, apoptosis, and inflammation in podocytes exposed to high glucose (41–43). • Promotion of autophagy by regulating Sirt1/glycogen synthase kinase 3β and Sirt1/NF-κB pathways reduces podocytes injury in diabetes (22, 44, 45). • Promotion of autophagy by inhibiting AMPK/mTOR pathway prevents diabetic podocytes injury (46–48). • Inhibition of autophagy by activating liver X receptor aggravates podocytes injury in diabetes (49). • Progranulin facilitates mitophagy and mitochondrial homeostasis *via* Sirt1-PGC-1α/FoxO1 signaling to prevent podocytes injury in DN (50).
Renal tubular epithelial cells	• High glucose-induced lipophagy deficiency in tubular cells causes ectopic lipid accumulation-associated kidney damage, which is relieved by promoting autophagy (29). • Smad family member 3 directly binds to the 3’ untranslated region of transcription factor EB and suppresses lysosome biogenesis to inhibit autophagy in tubular epithelial cells in DN (30). • Inhibition of autophagy by miR-22 targeting phosphatase and tensin homolog and miR-155-5p targeting Sirt1 induces renal tubular fibrosis in DN (32). • Promotion of autophagy by up-regulating AMPK pathway improves mitochondrial health (11, 51) and reduces fibrosis (52, 53) in renal tubular epithelial cells to reduce DN. • Autophagy causes the degradation of AGEs by up-regulation of lysosomal biogenesis and function in tubular epithelial cells to reduce DN (54). • Promotion of autophagy by inhibiting mTOR pathway counteracts high glucose-induced injury in tubular epithelial cells (55).
Glomerular mesangial cells	• Promotion of autophagy by activating AMPK/Sirt1 pathway (28, 56) or by Sirt1/NF-κB pathway (33) relieves high glucose-induced injury in glomerular mesangial cells. • Activation of Akt/mTOR pathway inhibits autophagy and accelerates inflammation and fibrosis in high glucose-treated glomerular mesangial cells (57, 58).
Glomerular endothelial cells	• Inhibition of AGE/RAGE axis restores the disturbed autophagy to alleviate glomerular endothelial permeability in DN (59). • Autophagy deficiency accompanying oxidative stress and apoptosis in high glucose-cultured glomerular endothelial cells is associated with CaMKKβ-LKB1-AMPK pathway (60). • Promotion of autophagy by inhibiting miR-34a/Atg4b pathway in glomerular endothelial cells relieves diabetic kidney damage (61).

DN, diabetic nephropathy; Sirt1, silent information regulator of transcription 1; AMPK, adenosine 5’-monophosphate-activated protein kinase; Akt, protein kinase B; mTOR, mammalian target of rapamycin; Bcl-2, B-cell lymphoma-2; NF-κB, nuclear factor kappa-B; PGC-1α, peroxisome proliferator-activated receptor-gamma coactivator-1α; FoxO1, forkhead box O1; AGEs, advanced glycation end-products; CAMKKβ, calcium/calmodulin-dependent protein kinase kinase β; LKB1, liver kinase B1; Atg, autophagy-related gene.

### 3.1 Podocytes

Podocytes, highly differentiated epithelial cells with a limited capacity for proliferation, tightly attach to the GBM (62) and work as an important part of the glomerular filtration barrier (GFB) (63, 64). The damage and apoptosis of podocytes can destroy the integrity of the GFB (31), leading to proteinuria, renal lesions, and finally DN (7, 8, 65).

A high level of autophagy in podocytes is necessary to keep the physiological function (8, 39), which is regulated by the adenosine 5’-monophosphate-activated protein kinase (AMPK) pathway rather than the mammalian target of rapamycin (mTOR) (66). The impairment of autophagy in diabetic podocytes as evidenced by the decreased expression of autophagy-related proteins (beclin1, LC3II/I, Atg12, Atg7, etc.) and the accumulation of the autophagic substrate p62 (40, 67) exacerbates the loss of podocytes with the help of the increased cellular lipid accumulation, oxidative stress, and inflammation (11, 41). Knockout of the Atg5 in podocytes has been reported to cause glomerular lesions accompanied by podocyte loss and albuminuria (68). These findings imply that the shortage of autophagy mediates podocyte damage in diabetes (22). It is interesting to note that the increased autophagosomes in high glucose-treated podocytes was not consistent with the impaired autophagy in the diabetic rat kidney characterized by glomerular hypertrophy, renal tubular expansion, and mesangial cell proliferation (44). To further clarify whether the rise in autophagosomes is caused by autophagy induction or the obstructed fusion of autophagosomes and lysosomes, the fusion inhibitor such as chloroquine can be adopted or the colocalization of LC3 and lysosomes need to be explored. In addition, this contradiction in different diabetic kidney models might be related to the different roles of autophagy in each stage of diabetes (3).

Nutrient signaling pathways are involved in the disorder of autophagy in diabetic podocytes. Increased mTOR activity and decreased expression of AMPK and silent information regulator of transcription 1 (Sirt1) in diabetes can inhibit autophagy to aggravate cellular dysfunction and the progression of DN (69, 70). The silence of AMPK or Sirt1 was reported to inhibit autophagy and promote the loss of podocyte function in a high glucose environment (12, 42, 43). Furthermore, the up-regulation AMPK/mTOR signaling pathway-mediated autophagy prevents the loss of podocyte markers (nephrin, podocin) and ameliorates diabetic kidney injury (46–48). Liver X receptor and high mobility group box 1 also induce podocyte injury by altering autophagy through the nutrient-sensing signal pathway (34, 49).

### 3.2 Renal tubular epithelial cells

The enhancement of autophagy in proximal tubular epithelial cells (PTECs) in response to multiple stresses such as ischemia and nephrotoxic medications has been reported to protect the kidney (71). Morphological alterations including hypertrophy, hyperplasia and epithelial-mesenchymal transition (EMT) in RTECs, especially in PTECs, primarily owing to the shortage of autophagy in diabetes, are regarded as an early sign of DN, which can easily cause renal dysfunction and even ESRD if not corrected in time (51, 72, 73).

It is noteworthy that the interaction of autophagy with EMT in RTECs is complicated, various factors and signaling pathways are associated with the effect of autophagy-related EMT on the progression of DN (33, 74). The role of rapamycin in reducing profibrotic cytokines, fibroblast proliferation, tubulointerstitial inflammation, and EMT confirms that mTOR-regulated autophagy is necessary for EMT in diabetic RTECs (69, 75). Interestingly, hyperglycemia-induced miR-22 promotes EMT by suppressing autophagy *via* targeting phosphatase and tensin homolog/protein kinase B (Akt)/mTOR signaling pathway, which suggests that targeting miRNA may be a promising therapeutic approach in preventing DN (32). Recently, mesenchymal stem cell-derived exosomes was reported to activate autophagy to inhibit transforming growth factor-β (TGF-β)-induced EMT progression in RTECs (76). Thus, the role of exosomes on the EMT in diabetic RTECs is worth further investigation.

In the presence of diabetes, carbonyl compounds created by advanced glycation end-products (AGEs) are filtered by the glomerulus and then reabsorbed by the proximal tubule, easily resulting in tubular toxicity (77, 78). Through interaction with the receptor for AGEs (RAGE), accumulation of AGEs triggers various abnormal cellular cascades like oxidative stress, inflammation, and apoptosis and inhibits the protective effect of autophagy in the diabetic kidney (79). The impairment of the autophagy-lysosomal pathway in diabetes promotes the accumulation of AGEs and the excessive AGEs aggravates lysosomal dysfunction, thus forming positive feedback to allow tubulointerstitial inflammation and fibrosis, which might be crucial to the development of DN (17, 80). Inhibiting AGEs/RAGE signaling is reported to restore the disturbed autophagy in glomerular endothelial cells and attenuate DN (59). It is said that AGEs can enhance the expression of profibrotic molecules linked to EMT and ER stress in the human renal tubular epithelial cell line to gradually render renal fibrosis (81), which is prevented by the enhancement of autophagy in RTECs (54). Therefore, the specific role of the AGEs/RAGE axis in DN is worthy of exploring.

### 3.3 Glomerular mesangial cells

Proliferation and hypertrophy in GMCs and mesangial expansion manifested as excess extracellular matrix (ECM) derived from GMCs are two pathological characteristics of DN, which lead to glomerulosclerosis and tubulointerstitial fibrosis (82, 83). Hyperglycemia, AGEs, and ROS all effectively activate TGF-β to cause ECM accumulation both in Smad-dependent and -independent pathways (84–86), which can be reversed by the up-regulation of autophagy (33, 57).

Sirt1 has been revealed to inhibit ECM accumulation in high glucose-treated GMCs *via* enhancing autophagy (33) and blocking mTOR-suppressed autophagy has also been documented to effectively reduce inflammation, proliferation, and fibrosis in diabetic GMCs (15, 28, 57). All of the above indicate that autophagy is important for maintaining the structural and functional integrity of GMCs to resist DN.

### 3.4 Glomerular endothelial cells

GEnCs, the first barrier of glomerular filtration, are vulnerable to hyperglycemia. The abnormal structure manifested as endothelial glycocalyx and endothelial-mesenchymal transition usually occur in the early stage of DN (87). Severe damage to the glomerular endothelium owing to autophagy reduction has been reported in endothelial-specific autophagy-deficient mice and Atg16L-knockdown GEnCs (88, 89). In addition, activation of calcium/calmodulin-dependent protein kinase kinase β (CAMKKβ)/liver kinase B1 (LKB1)/AMPK signaling (60) and inhibition of miR-34a/Atg4b signaling (61) promote autophagy in GEnCs to attenuate DN. It is well established that the interplay of podocytes, GEnCs, and GMCs is key to keep the integrity of the GFB and the pathological alteration in one component evidently affects the other two (87, 90, 91). These results imply that appropriate autophagy in GEnCs can minimize DN by preserving glomerular structural integrity.

## 4 Autophagic pathways in DN

**Figure 2:**
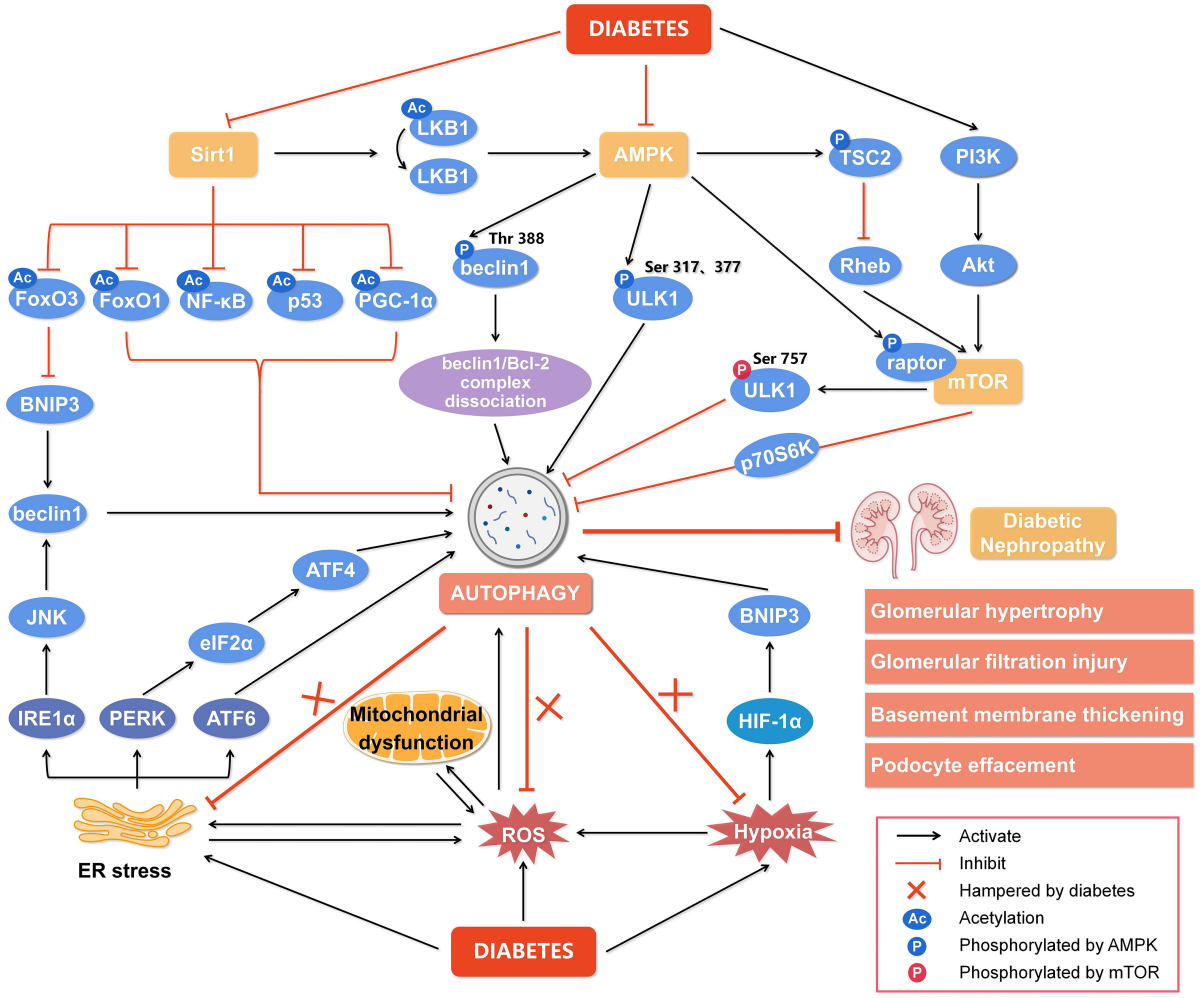
Regulation of autophagy during diabetic nephropathy. Hyperglycemia is considered a state of overnutrition, leading to over-activation of the mammalian target of rapamycin (mTOR) and inhibition of adenosine 5’-monophosphate-activated protein kinase (AMPK) and silent information regulator of transcription 1 (Sirt1). The activated mTOR inhibits autophagy by blocking unc-51-like kinase 1 (ULK1) activation by AMPK and its downstream target phosphoprotein 70 ribosomal protein S6 kinase (p70S6K). The inhibition of AMPK blocks the dissociation of the beclin1/Bcl-2 (B-cell lymphoma-2) complex and the phosphorylation of ULK1, while promotes mTOR activity to reduce autophagy. The inactivated Sirt1 reduces the deacetylation of several target genes like forkhead box O3 (FoxO3), FoxO1, nuclear factor kappa-B (NF-κB), p53, and peroxisome proliferator-activated receptor-gamma coactivator-1α (PGC-1α) to inhibit autophagy. In addition, other cellular events, including reactive oxygen species (ROS), endoplasmic reticulum (ER) stress, and hypoxia, can also regulate autophagy to affect the development of diabetic nephropathy. Hypoxia-inducible factor 1α (HIF-1α) induced by hypoxia promotes the transcription of Bcl-2/adenovirus E1V19-kDa interacting protein 3 (BNIP3) and induces autophagy. ER stress enhances the expression of ER membrane proteins like protein kinase RNA-like ER kinase (PERK), inositol-requiring enzyme 1α (IRE1α), and activating transcription factor 6 (ATF6), leading to autophagy. In addition, autophagy under ER stress may be associated with the signaling pathway of PERK/α-subunit of eukaryotic initiation factor 2 (eIF2α)/ATF4 and IRE1α/c-Jun N-terminal kinase (JNK)/beclin1. Significantly, the endogenous autophagy induced by ER stress, oxidative stress and hypoxia in diabetes is hampered, which aggravates the progression of diabetic nephropathy. Thus, impaired autophagy accelerates the progression of diabetic nephropathy, resulting in a series of renal pathological damages. Rheb, ras homolog enriched in brain; PI3K, class III phosphatidylinositol-3-kinase; Akt, protein kinase B.

Autophagy in eukaryotic cells is tightly regulated to adapt or counteract cellular stresses through multiple signaling pathways (17) because both insufficient and excessive autophagy are harmful (92). Nutrient-sensing pathways including AMPK, mTOR, and Sirt1 are well-recognized to regulate autophagy in diabetic complications (10). Moreover, various cellular stresses such as ROS, ER stress, and hypoxia are involved in pathogenic autophagy in DN (**Figure 2**) (93). Thus, autophagy in the development of DN is precisely regulated.

### 4.1 Nutrient-sensing pathways

#### 4.1.1 mTOR pathway

Rapamycin-sensitive type of mTOR (mTORC1), a master inhibitor of autophagy, is inhibited by starvation to reduce the phosphorylation of ULK1 at Ser757, which frees ULK1 to be activated by AMPK and then initiates autophagy to provide nutrients for the cell’s use by degrading the captured cytoplasmic components (94, 95). mTOR is over-mobilized in the diabetic kidney to promote the inflammatory response and exacerbate renal impairment (96, 97), which is reversed by rapamycin (98). In addition, the mTOR signaling pathway can be activated by vascular endothelial growth factor *via* PI3K/Akt cascade, which suppresses autophagy *via* phosphorylating its downstream phosphoprotein 70 ribosomal protein S6 kinase (p70S6K) and exacerbates DN (99, 100). All of these suggest that the overactivation of the mTOR pathway is extremely detrimental to the development of DN (101, 102). Numerous studies have demonstrated the critical role that long noncoding RNAs (LncRNAs) play in the pathophysiology of DN (103). LncRNAs potently affect the pathological alteration in the diabetic kidney by inhibiting the autophagy-related Akt/mTOR pathway, which has been supported by growing evidence that LncRNA silencing sperm-associated antigen 5 antisense RNA1 promotes hyperglycemia-induced injury in podocytes targeting Akt/mTOR signaling (37), and LncRNA nuclear enriched abundant transcript 1 accelerates (58), whereas LncRNA SOX2 overlapping transcript inhibits (15), proliferation and fibrosis in diabetic GMCs *via* modulating Akt/mTOR signaling-related autophagy. Thus, the effect of LncRNAs is diversified depending on the type of LncRNAs in the development of DN though the same target of Akt/mTOR signaling-related autophagy may be involved.

#### 4.1.2 AMPK pathway

AMPK belongs to the serine/threonine protein kinase family and is composed of the catalytic subunit α and the regulatory subunits β and γ (104). The phosphorylation of the threonine 172 (Thr172) site on the subunit α is necessary for the activation of AMPK (105). AMPK is regulated by the AMP/ATP ratio as an energy sensor (3). Under harmful conditions like hunger and hypoxia, the ratio of AMP/ATP ratio rises and renders AMP binding to the subunit γ of AMPK, which promotes Thr172 phosphorylation by LKB1 (106). In addition, AMPK is even activated by CAMKKβ and TGF-β-activated kinase by the action of hormones, drugs, or proinflammatory cytokines (106, 107) to trigger autophagy for keeping cellular energy homeostasis under starvation.

It has been shown that AMPK and autophagy are deactivated in the diabetic kidney accompanied by proteinuria and renal pathological alterations (11, 45, 56, 108). As shown in **Figure 2**, AMPK can phosphorylate ULK1 at Ser317 and Ser377 to directly initiate autophagy (109, 110) or indirectly promote autophagy by blocking mTORC1 to release ULK1 through phosphorylating tuberous sclerosis complex 2 (TSC2) and raptor, the critical mTORC1-binding subunit (111), which benefits to hinder the progression of DN (112). In addition, AMPK activates Sirt1 by increasing cellular NAD^+^ levels (56) or phosphorylating and redistributing glyceraldehyde 3-phosphate dehydrogenase into the nucleus to free Sirt1 (111), which promotes autophagy and alleviates DN (28, 56). AMPK can promote the dissociation of the beclin1/B-cell lymphoma-2 (Bcl-2) complex *via* phosphorylating beclin1 at Thr388 to initiate autophagy (113). Thus, AMPK-regulated autophagy is key to the development of DN and AMPK may be a promising target for preventing DN.

#### 4.1.3 Sirt1 pathway

Sirt1, the most widely studied NAD-dependent deacetylase in the Sirtuin family (114, 115), is highly expressed in renal tubular cells and podocytes (115) and has been reported to attenuate diabetic kidney disease by reducing the phosphorylation and acetylation levels of NF-κB and signal transducer and activator of transcription 3 (33, 44, 116). In addition, Sirt1 reduces acetylation or phosphorylation of several target genes such as AMPK, forkhead box O1 (FoxO1), p53, and peroxisome proliferator-activated receptor-gamma coactivator-1α (PGC-1α) to enhance autophagy (**Figure 2**) (28, 50, 117). As a positive regulator of autophagy, Sirt1 has been revealed to up-regulate Bcl-2/adenovirus E1V19-kDa interacting protein 3 (BNIP3) by deacetylating the transcription factor FoxO3 to enhance autophagy and inhibit DN (118, 119). LKB1 deacetylated by Sirt1 activates AMPK to enhance autophagy (120, 121). In addition, deacetylation of p53 by Sirt1 potently activates AMPK-dependent autophagy to ameliorate DN (12) and this protective effect of Sirt1 against DN is inhibited by several miRNAs including miR-135a-5p (122), miR-138 (65), miR-150-5p (12), miR-155-5p (123), and miR-217 (124) targeting the 3’ untranslated region of Sirt1. The relationship between miRNAs and Sirt1 is complicated in the progression of DN and more efforts are needed to clarify the underlying mechanism by which Sirt1-regulated autophagy prevents DN.

### 4.2 Cellular stress signaling

#### 4.2.1 Oxidative stress

Excessive production of ROS and/or reactive nitrogen species beyond the endogenous scavenging capacity leads to oxidative stress. Oxidative damage of cellular lipids, proteins, nucleic acids, and carbohydrates breaks the structural integrity and results in physiological dysfunction (125). Oxidative stress induced by hyperglycemia through *de novo* ROS generation and suppression of the antioxidant defense system promotes mitochondria swelling, cristae breakage, and mitochondrial disintegration in the diabetic kidney, which can be reversed by the enhancing autophagy to eliminate damaged mitochondria (126).

It should be noted that autophagy and oxidative stress are interactive. ROS are reported as an early inducer for autophagy initiation and execution, which may be a crucial adaptive response to reduce oxidative stress and obtain the nutrient for reuse through autophagy-dependent degrading oxidative damaged cellular components (127). On the contrary, oxidative modification of key upstream autophagy regulators and autophagy core proteins including AMPK, Sirt1, Atg4, and Parkin impair autophagy (128). Thus, oxidative stress affects autophagy in the development of DN as a two-edged sword and antioxidant therapy may protect the kidney against diabetes through activating autophagy. This notion has been supported by some evidence that antioxidant compounds derived from plants such as betulinic acid, ursolic acid, genistein, and luteolin effectively attenuate the kidney injury induced by diabetes or poisons by promoting autophagy (38, 129–132).

#### 4.2.2 Endoplasmic reticulum stress

The accumulation of unfolded or misfolded proteins in the ER lumen leads to ER stress which is evident in DN (24, 133). Overproduction of ROS due to chronic hyperglycemia disrupts intracellular Ca^2+^ homeostasis and oxidation of ER-resident proteins to trigger ER stress, in turn, hyperactivates the oxidative folding machinery to correct improper disulfide bonds, further producing ROS (134, 135). This vicious cycle leads to the disruption of cellular homeostasis (**Figure 2**). Emerging evidence suggests that autophagy is linked to the unfolded protein response (UPR) to relieve ER stress by clearing misfolded proteins (24, 136, 137). Under ER stress, the UPR is triggered by three protein sensors, protein kinase RNA-like ER kinase (PERK), inositol-requiring enzyme 1α (IRE1α), and activating transcription factor 6 (ATF6) after accumulation of misfolded proteins (24). As shown in **Figure 2**, all these three sensors of the UPR under ER stress can induce autophagy *via* activating signaling pathways of PERK/α-subunit of eukaryotic initiation factor 2/activating transcription factor 4 (PERK/eIF2α/ATF4) (138), IRE1α/c-Jun N-terminal kinase (JNK)/beclin1 and ATF6 (24, 139). The negative regulator of autophagy mTOR in diabetic PTECs is activated accompanying the increase of ER stress (140) and activating autophagy by Jujuboside A potently attenuates ER stress and cell death in the diabetic kidney (141). The autophagy in the kidney is usually inhibited under diabetic status (142, 143), which is reversed by the ER stress inhibitors salubrinal and tauroursodeoxycholic acid (143). Since ER stress inhibitors such as tauroursodeoxycholic acid, ursodeoxycholic acid, and 4-phenylbutyrate potently rescue diabetic renal tubules and podocytes (144, 145), investigating in detail the interaction between ER stress and autophagy in the progression of DN is promising.

#### 4.2.3 Hypoxia stress

Kidney hypoxia, preceding the onset of albuminuria (146) and correlating with reduced glomerular filtration rate, runs through the whole stage of DN owing to the limited capacity of enhancing renal plasma flow and oxygen delivery (147). Hypoxia-inducible factor (HIF) is key to adaptively maintain cellular homeostasis by transcriptionally activating the expression of several target genes in response to hypoxia (148, 149).

Accumulating evidence shows that hypoxia is an important pathogenic factor for DN. Deficiency of HIF-1α has been reported to aggravate renal dysfunction (150), while up-regulation of HIF-1α effectively enhances autophagy to mitigate DN, which may associate with the increased expression of Sirt1, FoxO3, and BNIP3 (119, 151, 152). Recent studies demonstrate that up-regulation of sestrin2 by HIF-1α is involved in hypoxia-related diseases (153), which may modulate AMPK and mTORC1-dependent autophagy to reduce the production of ROS and attenuate DN (154, 155). Thus, the deteriorating effect of hypoxia on the diabetic kidney is not ignored and HIF-1α-related autophagy may be a potential target for treating DN.

## 5 Therapeutic strategies targeting autophagy for DN

The symptomatic treatment for DN usually includes glycemic control, reducing albuminuria, and blocking RAS with the usage of angiotensin-converting enzyme inhibitors (ACEI) and angiotensin receptor antagonists (ARB) (156, 157). New hypoglycemic agents such as sodium-glucose cotransporter 2 (SGLT2) inhibitors, glucagon-like peptide 1 receptor (GLP-1R) agonists, and dipeptidyl peptidase-4 (DPP-4) inhibitors have been shown to protect the diabetic kidney *via* modulating autophagy (**Table 2**).

**Table 2:** Agents targeting autophagy for diabetic nephropathy.

Agent		Experimental models	Effect for pathology of renal injuries	Reference
SGLT2 inhibitors	Dapagliflozin	Human PTECs (HK-2 cell) exposed to high glucose	Ameliorating autophagic flux and reducing inflammation by inhibiting NF-κB pathway through AMPK activation.	(55)
		HFD-induced prediabetic rats	Reducing oxidative stress, ER stress, inflammation, and apoptosis and up-regulating autophagy.	(158, 159)
	Empagliflozin	STZ-induced diabetic mice; Human PTECs (HKC-8) exposed to high glucose	Enhancing autophagy and mitochondrial function to reverse renal morphological changes.	(51)
		db/db mice	Reactivating autophagy and improving glomerular morphology.	(160)
GLP-1R agonists	Liraglutide	Zucker diabetic fatty rats; Human PTECs (HKC-8) exposed to AGEs	Activating autophagy and reducing oxidative stress *via* AMPK/mTOR pathway.	(161)
DPP-4 inhibitors	Linagliptin	db/db mice	Reactivating glomerular autophagy and improving glomerular morphology.	(160)
Metformin		HFD/STZ-induced diabetic rats; Renal mesangial cells exposed to high glucose	Enhancing autophagy *via* AMPK/Sirt1-FoxO1 pathway and alleviating oxidative stress.	(28)
		HFD/STZ-induced diabetic rats; RTECs exposed to high glucose	Attenuating renal fibrosis *via* activating AMPK-induced autophagy and suppressing EMT.	(52)
Rapamycin		STZ-induced diabetic rats	Enhancing autophagy by inhibiting mTOR and improving renal function.	(14)
		db/db mice	Reducing fat deposition, pathological changes and renal dysfunctions *via* inhibiting mTOR.	(98)
Other candidate drugs	Melatonin	STZ-induced diabetic rats; RTECs (NRK52E) exposed to high glucose	Enhancing autophagy and mitochondrial biogenesis *via* activating the AMPK/Sirt1 axis.	(11)
	Resveratrol	db/db mice; Human podocytes exposed to high glucose	Activating autophagy and attenuating apoptosis through the suppression of miR-383-5p.	(35)
		STZ-induced diabetic rats	Normalizing lipid metabolism by inducing AMPK/mTOR-mediated autophagy.	(162)
	Vitamin D analogs	STZ-induced diabetic mice; Human PTECs (HK-2 cell) exposed to high glucose	Restoring defective autophagy through CAMKKβ-AMPK pathway.	(53)

SGLT2, sodium-glucose cotransporter 2; PTECs, proximal tubular epithelial cells; NF-κB, nuclear factor kappa-B; AMPK, adenosine 5’-monophosphate-activated protein kinase; HFD, high-fat diet; ER, endoplasmic reticulum; STZ, streptozotocin; GLP-1R, glucagon-like peptide 1 receptor; AGEs, advanced glycation end-products; mTOR, mammalian target of rapamycin; DPP-4, dipeptidyl peptidase-4; FoxO1, forkhead box O1; EMT, epithelial-mesenchymal transition; CAMKKβ, calcium/calmodulin-dependent protein kinase kinase β.

Inhibiting SGLT2, located on the lumen surface of PTECs, potently lowers blood glucose by reducing the reabsorption of glucose (163). SGLT2 inhibitors empagliflozin and dapagliflozin have been shown to enhance autophagy depending on AMPK/mTOR pathway to attenuate diabetic kidney injury (51, 55, 158). Additionally, the progression of renal complications in pre-diabetes is slowed by dapagliflozin through the suppression of renal inflammation, ER stress, and apoptosis (159). Although the commercially available SGLT2 inhibitors including empagliflozin, dapagliflozin, and canagliflozin have been used in clinics (147), the protective effect against DN has not been fully elucidated (164).

Liraglutide, a GLP-1R analogue to lower blood glucose, has been shown to significantly improve the prognosis for DN (165), which may be related to reducing apoptosis and oxidative stress through promoting AMPK-regulated autophagy (161, 166). DPP-4 inhibitor linagliptin not only hinders the degradation of endogenous GLP-1 to lower blood glucose, but also alleviates mesangial expansion, podocyte foot process effacement, and albuminuria excretion in the diabetic kidney by reactivating autophagy (160). Additionally, the hypoglycemic agent metformin was reported to mitigate tubulointerstitial fibrosis and oxidative stress in diabetes by enhancing autophagy through AMPK/Sirt1/FoxO1 pathway (28, 52). Rapamycin has been shown to improve the short-term pathological alterations in DN by enhancing autophagy by blocking the mTORC1/ULK1 pathway (9). However, the serious side effect of rapamycin limits its use in long-term clinical treatment (75). Animal studies showed that melatonin, resveratrol, and vitamin D analogs prevent DN by modulating AMPK-regulated autophagy (11, 35, 53, 162), which may be the candidate drug for treating DN in the clinic.

Recently, exosome is becoming a promising therapeutic target for DN treatment (167). Exosome, as a kind of extracellular vesicles, is involved in intercellular communication by carrying various biomolecules and may be a novel biomarker for evaluating the progression of DN (168, 169). MiRNAs contained in the exosome derived from different cells attenuate high glucose-induced renal cell injury by promoting autophagy (36, 170, 171). Additionally, mesenchymal stem cell-derived exosomes induce autophagy *via* inhibiting mTOR to attenuate diabetic renal fibrosis (172). It is evident that the more we understand DN, the more we can do about DN. Exosome therapy combined with autophagy regulation may be promising for treating DN.

## 6 Conclusion

The significant increase in the incidence of diabetes has become a serious worldwide health issue. The high mortality of diabetes is strongly correlated with DN and the subsequent ESRD. Due to the complexity and diversity of the pathogenesis of DN, both rigorous control of blood glucose and cholesterol and blocking RAS with the usage of ACEI and ARB do not improve the endpoint of DN. The role of autophagy in the progression of DN sheds light on treating DN and how to keep the balance of autophagy in the diabetic kidney may be a new direction for prevention and management of DN though more efforts should be paid to exploring the precise regulation of autophagy in DN.

## Author contributions

Y-PH wrote the manuscript. Y-PH, L-JL, J-LY, M-YC, X-FM, X-RZ, and L-BQ designed the figures and edited the manuscript. X-RZ and L-BQ supervised the writing. All authors contributed to the article and approved the submitted version.

## Glossary

**Table:**

ACEI	angiotensin-converting enzyme inhibitors
AGEs	advanced glycation end-products
Akt	protein kinase B
Atg	autophagy-related gene
AMPK	adenosine 5’-monophosphate-activated protein kinase
ARB	angiotensin receptor antagonists
ATF	activating transcription factor
Bcl-2	B-cell lymphoma-2
BNIP3	Bcl-2/adenovirus E1V19-kDa interacting protein 3
CAMKKβ	calcium/calmodulin-dependent protein kinase kinase β
DN	diabetic nephropathy
DPP-4	dipeptidyl peptidase-4
ECM	extracellular matrix
EMT	epithelial-mesenchymal transition
ER	endoplasmic reticulum
ESRD	end-stage renal disease
eIF2α	α-subunit of eukaryotic initiation factor 2
FoxO	forkhead box O
GBM	glomerular basement membrane
GEnCs	glomerular endothelial cells
GFB	glomerular filtration barrier
GLP-1R	glucagon-like peptide 1 receptor
GMCs	glomerular mesangial cells
HFD	high-fat diet
HIF	hypoxia-inducible factor
IDF	International Diabetes Federation
IRE1α	inositol-requiring enzyme 1α
JNK	c-Jun N-terminal kinase
LC3	microtubule-associated protein 1A/1B-light chain 3
LKB1	liver kinase B1
LncRNAs	long noncoding RNAs
mTOR	mammalian target of rapamycin
NF-κB	nuclear factor kappa-B
PERK	protein kinase RNA-like ER kinase
p70S6K	phosphoprotein 70 ribosomal protein S6 kinase
PGC-1α	peroxisome proliferator-activated receptor-gamma coactivator-1α
PI3K	class III phosphatidylinositol-3-kinase
PTECs	proximal tubular epithelial cells
RAS	renin-angiotensin system
RAGE	receptor for advanced glycation end-products
Rheb	ras homolog enriched in brain
ROS	reactive oxygen species
RTECs	renal tubular epithelial cells
SGLT2	sodium-glucose cotransporter 2
Sirt1	silent information regulator of transcription 1
STZ	streptozotocin
TGF-β	transforming growth factor-β
TSC2	tuberous sclerosis complex 2
ULK1	unc-51-like kinase 1
UPR	unfolded protein response
